# SRF is a nonhistone methylation target of KDM2B and SET7 in the regulation of skeletal muscle differentiation

**DOI:** 10.1038/s12276-021-00564-4

**Published:** 2021-02-09

**Authors:** Duk-Hwa Kwon, Joo-Young Kang, Hosouk Joung, Ji-Young Kim, Anna Jeong, Hyun-Ki Min, Sera Shin, Yun-Gyeong Lee, Young-Kook Kim, Sang-Beom Seo, Hyun Kook

**Affiliations:** 1 Department of Pharmacology, Chonnam National University Medical School, Hwasun, Republic of Korea; 2 Vascular Remodeling Research Center, Chonnam National University Medical School, Hwasun, Republic of Korea; 3 Department of Life Sciences, College of Natural Sciences, Chung-Ang University, Seoul, Republic of Korea; 4 Department of Biochemistry, Chonnam National University Medical School, Hwasun, Republic of Korea

**Keywords:** Methylation, Differentiation

## Abstract

The demethylation of histone lysine residues, one of the most important modifications in transcriptional regulation, is associated with various physiological states. KDM2B is a demethylase of histones H3K4, H3K36, and H3K79 and is associated with the repression of transcription. Here, we present a novel mechanism by which KDM2B demethylates serum response factor (SRF) K165 to negatively regulate muscle differentiation, which is counteracted by the histone methyltransferase SET7. We show that KDM2B inhibited skeletal muscle differentiation by inhibiting the transcription of SRF-dependent genes. Both KDM2B and SET7 regulated the balance of SRF K165 methylation. SRF K165 methylation was required for the transcriptional activation of SRF and for the promoter occupancy of SRF-dependent genes. SET7 inhibitors blocked muscle cell differentiation. Taken together, these data indicate that SRF is a nonhistone target of KDM2B and that the methylation balance of SRF as maintained by KDM2B and SET7 plays an important role in muscle cell differentiation.

## Muscles: competing enzymes control cell development

The balance between the opposing actions of two enzymes that, respectively, inhibit or activate the same gene-regulating protein plays a key role in the differentiation of skeletal muscle cells. A South Korean team led by Hyun Koon at Chonnam National University, Hwasun, and Sang-Beom Seo at Chung-Ang University, Seoul, studied the effect of the enzymes on cultured human muscle-forming cells. One enzyme, KDM2B, inhibits the differentiation of the cells by removing methyl groups (CH_3_) from a protein called serum response factor (SRF). A second enzyme, SET7, activates differentiation by adding methyl groups to SRF. The SRF protein controls the activity of many genes required for cell differentiation. This work reveals SRF as a previously unidentified target for KDM2B, and provides new insights into skeletal muscle development in health and disease.

## Introduction

The amino acid residues of proteins are susceptible to diverse covalent modifications. These posttranslational modifications (PTMs) are involved in normal homeostasis and in pathologic conditions such as cancer^1,2^. Protein methylation, the addition of a methyl group to a target protein, is an important PTM and plays important roles in various human pathophysiologies^3–5^. Protein methylation is balanced by two sets of enzymes: methyltransferases and demethylases. Histone methylation occurs on the nitrogen-containing side chains of arginine or lysine. In contrast to arginine methylation, which is mediated by a series of proteins called PRDMs, which contain the PR (PRD1-BF1 and RIZ homology) domain, lysine is methylated by a family of methyltransferases with a SET [Su(var)3–9, enhancer-of-zeste and trithorax] domain^6,7^.

Serum response factor (SRF) is critical for cell survival and differentiation^1,5,8,9^. SRF directly binds to the serum response element (SRE) in the promoter of its target genes^8–10^. Many of the biological activities of SRF, such as DNA binding, self-dimerization, and interaction with other transcription factors, take place at the conserved MADS (MCM1, agamous, deficiens)-box domain^11,12^. In skeletal muscle cells, SRF functions as a transcription factor to induce SRF-dependent muscle-specific genes^8,9,13^. In addition to its role in DNA binding, the MADS-box recruits other transcription factors to form multicomponent regulatory complexes. We found that enhancer of polycomb1 (EPC1), a chromatin protein, directly binds to SRF to potentiate the activation of muscle regulatory factors (MRFs)^8^.

KDM2B, also known as JHDM1B/FBXL10, is an Fe(II)-dependent and alpha-ketoglutarate-dependent histone demethylase. It consists of multiple functional domains: an N-terminal JmjC domain, a CXXC zinc finger domain, a PHD domain, an F-box domain, and seven leucine-rich repeats. The JmjC domain catalyzes the demethylation of H3K4me3 and H3K36me2, leading to the transcriptional repression of target genes^14,15^. Recently, we discovered that KDM2B demethylates H3K79 and regulates transcriptional repression in a sirtuin-1-dependent manner^16^. Recent studies have shown that KDM2B is overexpressed in various types of cancers^17,18^. KDM2B promotes cancer cell proliferation and metastasis, cancer stem cell self-renewal, and drug resistance^17,18^. However, KDM2B can also decrease cancer cell proliferation by inhibiting the expression of oncogenes^17^. In addition to regulating cell proliferation and, thus, tumor biology, KDM2B as part of polycomb repressive complex 1 (PRC1) inhibits adipogenesis but in a demethylase-independent manner^19^. However, the role of KDM2B histone demethylase in myogenic differentiation remains unknown.

Here, we report a novel mechanism of KDM2B-mediated demethylation of K165 in SRF, which counteracts SET7-induced methylation. The methylation of SRF is required for the transcriptional activation of muscle-specific genes by altering its binding affinity for the SRE in the promoter of its target genes.

## Materials and methods

### Antibodies and reagents

Antibodies against SRF (sc-335), MyoD (sc-304), Set7/9 (sc-390823), MCK (sc-15164), skeletal α-actin (sc-58671), and GAPDH (sc-166574) were obtained from Santa Cruz Biotechnology (Santa Cruz, CA, USA). Antibodies against Flag (F1804), HA (H9658), GFP (G1544), and actin (A2066) were purchased from Sigma-Aldrich (St. Louis, MO, USA). Antibodies against histone H3 (tri-methylated K4, ab8580), histone H3 (dimethylated K36, ab9049), pan-methylated lysine (ab7315), and lamin B1 (ab16048) were obtained from Abcam (Cambridge, UK). Anti-KDM2B/JHDM1B antibody (09–864) was obtained from EMD Millipore Corp. (Billerica, MA, USA). Anti-MHC antibody (MF-20) was obtained from DSHB (Iowa City, IA, USA). Anti-V5 antibody (46–0705) was obtained from Invitrogen (Carlsbad, CA, USA). SRF-derived peptides (1~5, K154, K163, K165, and K165A) were synthesized by Peptron (Daejeon, South Korea). Sinefungin and (R)-PFI-2 were purchased from Sigma-Aldrich (St. Louis, MO, USA).

### Plasmid constructs and transfection

*pCGN-SRF-HA* was kindly provided by Prof. Jonathan A. Epstein (University of Pennsylvania, Philadelphia, PA, USA). *pCMV-3xFlag-KDM2B* was described previously^16^. *pHM6-HA-MyoD* was obtained from Prof. Young Kyu Ko (College of Life Sciences and Biotechnology, Korea University, Seoul, South Korea). The three truncation mutants of SRF were constructed: one contains the SRF A domain (amino acids 1 to 133), one contains the SRF B domain (amino acids 133 to 222), and one contains the SRF C domain (amino acids 222 to 508). Mutant SRF was constructed by site-directed mutagenesis based on wild-type *pCGN-SRF-HA* (Cosmo Genetech, Seoul, South Korea). *pGL3-basic*-*myogenin-luciferase* and *pGL3*-*Mck-luciferase* were kind gifts from Prof. Da-Zhi Wang (University of North Carolina, NC). For the *Acta1* promoter-reporter assay, base pairs from −450 to +26 in the transcription start site mouse genomic DNA were amplified and subcloned into a *pGL3* basic vector.

C2C12 cells were transfected using Lipofectamine and Plus reagent (Invitrogen, Carlsbad, CA, USA) according to the manufacturer’s directions. Each recombinant expression vector was transiently transfected into HEK293T cells with PEI reagents according to the manufacturer’s instructions. An siRNA against KDM2B was obtained from Bioneer (Daejeon, South Korea). Cells were transfected with this siRNA (50 nM) using Lipofectamine RNAi MAX (Invitrogen, Carlsbad, CA, USA) according to the manufacturer’s instructions.

### Cell culture and subcellular fractionation

C2C12 cell lines were maintained in Dulbecco’s modified Eagle’s medium (DMEM) supplemented with 15% fetal bovine serum (FBS) and antibiotics. DMEM with 2% horse serum was used as the differentiation medium. HEK293T cells were maintained in DMEM supplemented with 10% FBS and antibiotics, as previously described. C2C12 cell survival was determined by 3-(4,5-dimethylthiazol-2-yl)-2,5-diphenyltetrazolium bromide (MTT) assay (Sigma-Aldrich M5655). After the addition of MTT to the medium, the optical density was determined at 570 nm using a microplate reader. To separate nuclear proteins, NE-PER nuclear and cytoplasmic extraction reagents (Thermo Fisher Scientific, Waltham, MA, USA, #78833) were used.

### RNA isolation and real-time quantitative PCR (q-PCR)

Total RNA was isolated from cells using NucleoSpin kits (Macherey-Nagel, Bethlehem, PA, USA). One microgram of total RNA was used for q-PCR analysis. cDNA was synthesized using ReverTra Ace cDNA synthesis kits (TOYOBO, Nipro, Osaka, Japan), and 1 μL of the cDNA synthesis reaction mixture was used with a Platinum SYBR Green kit from Qiagen (Hilden, Germany). q-PCR was performed using a Rotor-Gene Q Real-time PCR machine (Qiagen, Hilden, Germany). The oligonucleotides used in PCR were as follows: Kdm2b forward, 5′-AGCAGCTAAAACCTGGCAAA-3′, and reverse, 5′-GTGAGCTGGAACGTGACTGA-3′; Acta1 forward, 5′-GACCTCACTGACTACCTGATGAAA-3′, and reverse, 5′-CAGACTCCATACCGATAAAGGAAG-3′; myogenin forward, 5′-AGTACATTGAGCGCCTACAG-3′, and reverse, 5′-ACCCACCCTGACAGACAATC-3′; Mck forward, 5′-AGCAGCTCATTGATGACCAC-3′, and reverse, 5′-TCAAACTTGGGGTGCTTGCT-3′; Myod forward, 5’-TGCTCTGATGGCATGATGGA-3’, and reverse, 5’-CACTATGCTGGACAGGCAGT-3’; Set7 forward, 5’-TGAGGATGGAGGTGTTCTCC-3’, and reverse, 5’-TCTCCCGTCATCTCTCCATC-3’; and beta-actin forward, 5′-CACGATGGAGGGGCCGGACTCATC-3′, and reverse, 5′-TAAAGACCTCTATGCCAACACAGT-3′.

### Immunoblot analysis

Cell lysates were obtained from cells and tissues using RIPA buffer (R2002, Biosesang, Seongnam, South Korea) or 0.5% NP lysis buffer supplemented with protease inhibitors. Approximately 20–30 μg of the total lysates form cell lines or IP-eluted samples were separated on 10% or 12% SDS-PAGE gels and subsequently transferred to PVDF membranes (Millipore, Billerica, MA, USA). The membranes were blocked in 5% skim milk in TBST (0.05% Tween 20) buffer for 1 h at room temperature and then incubated overnight with the primary antibodies in 5% milk in TBST buffer at 4 °C. After washing in TBST 3× for 5 min each time, the membranes were incubated with the secondary antibodies, anti-mouse IgG and anti-rabbit IgG conjugated with horseradish peroxidase (HRP), for 1 h at room temperature. After washing in TBST 3× for 10 min each time, the membranes were incubated with ECL reagent (Millipore, Billerica, MA, USA). Analysis of western blots was conducted using a c300 system (Azure Biosystem, Inc., Dublin, CA, USA). In addition, mouse developmental skeletal muscle tissue blot (MW-102-d, Zyagen, San Diego, CA, USA) and mouse normal tissue blot II (1562; ProSci, San Diego, CA, USA) were used for western blotting under the same conditions.

### Protein immunoprecipitation (IP)

HEK293T and C2C12 cells were lysed in 0.5% NP40 lysis buffer supplemented with a protease inhibitor. Approximately 1–2 mg of the total lysate was incubated with 2 μg of antibody or IgG (Santa Cruz Biotechnology, Santa Cruz, CA, USA) coupled to protein A/G agarose beads (Santa Cruz Biotechnology, Santa Cruz, CA, USA) overnight at 4 °C. The beads were washed with ice-cold lysis buffer three times, and the proteins were then eluted from the beads with SDS buffer and subjected to SDS-PAGE.

### Luciferase reporter gene assay

For the luciferase assay, 293T and C2C12 cells were plated on 24-well plates and cultured for 24 h prior to transfection. The cells were transfected with plasmids containing *pCMV-beta-galactosidase*, which was used to normalize the luciferase assay. Forty-eight hours after transfection, the cells were washed with PBS, dissolved in reporter lysis buffer (Promega, Madison, WI, USA), and harvested by scraping. Lysate samples were mixed with luciferase assay reagent, and measurements were taken using a luminometer.

### Bacterial expression of GST-fusion proteins

SRF, SRF-K165A, and KDM2B_1–734_ were cloned into pGEX-4T-1 (GST tag vector) (GE Healthcare, Marlborough, MA, USA). SET7 was cloned into pGEX-4T-2 (GE Healthcare, Marlborough, MA, USA). The BL21 strain of *E. coli* was transformed with the target constructs. The transformants of SRF, SRF-K165A, and SET7 were grown in 2× YT medium (16 g/L tryptone, 10 g/L yeast extract, and 5 g/L NaCl) until the OD600 was ~0.6 and ten were induced with 0.02 mM IPTG at 37 °C for 4 h. In the case of the KDM2B_1–734_ transformants, induction was performed at 30 °C for 4 h. The bacteria were harvested, and the GST-tagged target protein was purified using glutathione Sepharose 4B (GE Healthcare, Marlborough, MA, USA) according to the manufacturer’s instructions.

### In vitro methyltransferase assay

Recombinant GST-SRF protein or synthetic SRF peptides were incubated for 3 h at 30 °C with GST-SET7 in the presence of 100 nCi of S-adenosyl-[methyl-^14^C]-L-methionine [^14^C-SAM] (Perkin Elmer, Waltham, MA, USA) in HMTase assay buffer (50 mM Tris–HCl [pH 8.5], 20 mM KCl, 10 mM MgCl_2_, 10 mM β-mercaptoethanol, and 1.25 M sucrose). The reaction products were separated by SDS-PAGE and analyzed with a phosphorimager (Bio-Rad, Irvine, CA, USA).

### In vitro demethylase assay

Recombinant GST-SRF protein or synthetic SRF peptides were incubated for 6 h at 37 °C with GST-KDM2B_1–734_ in demethylation assay buffer (20 mM Tris-HCl [pH 7.3], 150 mM NaCl, 1 mM a-ketoglutarate, 50 mM FeSO_4_, and 2 mM ascorbic acid) following ^14^C labeling using GST-SET7. The reaction products were separated by SDS-PAGE and analyzed with a phosphorimager.

### Scintillation counting

Synthetic SRF peptides were subjected to an in vitro methyltransferase assay using GST-SET7. The ^14^C-labeled peptides were transferred onto p81 filter paper (Millipore) and washed three times with 95% ethanol for 5 min each time at room temperature. The filters were allowed to air-dry, after which 2 mL of Ultima Gold (Perkin Elmer, Waltham, MA, USA) was added. ^14^C-SAM was then quantified using a scintillation counter.

To measure the remaining radioactivity after the in vitro demethylase assay, biotin-conjugated SRF peptides were ^14^C-labeled using GST-SET7, pulled down using streptavidin beads, and then incubated overnight at 37 °C with GST-KDM2B_1–734_ in demethylation assay buffer. The beads were washed, after which 1 mL of Ultima Gold was added, and ^14^C-SAM was quantified using a scintillation counter.

### Chromatin immunoprecipitation assay

Chromatin immunoprecipitation (ChIP) assays were conducted with an EpiQuik chromatin immunoprecipitation kit (EpiGentek, Farmingdale, NY, USA) according to the manufacturer’s protocol. Briefly, C2C12 cells were treated with 1% formaldehyde for 10 min to induce cross-linking between proteins and DNA that interact within intact chromatin. The cells were then sonicated to shear the chromatin in fragments between 100 and 500 bp. The sonicated chromatin was immunoprecipitated with anti-SRF, anti-H3K4me3, anti-H3K36me2, and anti-HA antibodies, while the negative control was immunoprecipitated with nonimmunized IgG. The oligonucleotides used for ChIP PCR were *SRE* proximal forward, 5′-AGTCCTCTCCTTCTTTGGTCAGT-3′, and reverse, 5′-TCCCCTTGCACAGGTTTTTAT-3′ and *SRE* distal forward, 5′-GGGCTTATTTTCCATCCCTACC-3′, and reverse, 5′-GTTTGAAAGGTCTCCCCAGTTC-3′.

### Gel shift assay

A nonradioactive LightShift chemiluminescent EMSA kit (Thermo Fisher Scientific, Waltham, MA, USA, #20148) was used to determine whether SRF binds to the putative SRF binding sites (*SREs*) in the Acta1 proximal promoter. The target oligonucleotide was labeled with biotin on the 3’ end using a Biotin 3′ End DNA labeling kit (Thermo Fisher Scientific, Waltham, MA, USA, #89818). The oligonucleotides used as probes or competitors in the gel shift assays were end-labeled at the 3′ end with biotin. The sequences were as follows: *SRE* near sense, 5′-GACACCCAAATATGGCTTGG-3′, and antisense, 5′-CCAAGCCATATTTGGGTGTC-3′, and *SRE* far sense, 5′-AGAACCCATAAATGGGGTGC-3′, and antisense, 5′-GCACCCCATTTATGGGTTCT-3′. The complementary oligonucleotide pairs were annealed into double strands. Nuclear cell extracts were incubated for 30 min with biotin-labeled probes in binding buffer (included with the EMSA kit) at room temperature. An unlabeled oligonucleotide with the same sequence was used as the competitor. Supershift analysis was performed by adding anti-SRF antibody. Loading buffer was added to the reaction mixtures, and the binding reaction mixtures were subjected to electrophoresis on 5% PAGE gels in 0.5× TBE buffer at 100 V for 1 h. After electrophoresis, the bound reactants were then transferred onto Biodyne B nylon membranes (Thermo Fisher Scientific, Waltham, MA, USA, #77016) using 0.5× TBE for 1 h at 60 V. At the end of the transfer process, the transferred DNA-protein complexes were then cross-linked onto membranes using a hand-held UV lamp equipped with 254-nm bulbs that was set at a distance of approximately 0.5 cm from the membrane for an exposure time of 5–10 min. The blot was visualized with a c300 system (Azure Biosystem, Inc., Dublin, CA, USA).

### Immunofluorescence analysis and multinucleated cell counting

C2C12 cells were seeded for fusion or differentiation analysis in triplicate. The cells were fixed in 2% paraformaldehyde for 10 min at room temperature, permeabilized with 0.2% Triton for 5 min at room temperature, and blocked with 2–3% normal goat serum for 30 min at room temperature. Primary antibodies against MHC (DSHB, Iowa City, IA, USA) were diluted 1:50 in blocking buffer and incubated at room temperature for 2 h; the cells were then incubated with Alexa-conjugated secondary antibody (Molecular Probes, Invitrogen, Carlsbad, CA, USA). After washing, the nuclei were stained with DAPI (p36935, Invitrogen, Carlsbad, CA, USA). The stained cells were analyzed using a fluorescence microscope. Either the total number of nuclei or the number of nuclei within the MHC-positive myotubes was counted in 10 individual fields per well. The fusion index was determined by the following calculation: fusion index (%) = (number of nuclei within MHC-stained myotubes/total number of nuclei) ×100. All experiments were performed in triplicate.

### Statistical analysis

The data are presented as the means ± SEM. The data were analyzed using either unpaired Student’s *t* test or one-way analysis of variance, followed by Tukey’s honestly significant difference (HSD) multiple-comparison post hoc test. The statistical analysis was performed with PASW Statistics 21 (SPSS, IBM Company, Chicago, IL). Differences were considered to be significant when *p* < 0.05.

## Results

### KDM2B is expressed early in skeletal muscle differentiation

We first examined the distribution of KDM2B in the tissues of adult mice. KDM2B was highly expressed in the brain (Supplementary Fig. S1a), as previously reported^19^. To investigate the role of KDM2B in skeletal muscle, we first checked its expression using a C2C12 myoblast cell line. Differentiation medium (DM) reduced the expression of KDM2B in the C2C12 cells (Supplementary Fig. S1b). *Kdm2b* expression was also reduced, as evidenced by the quantitative RT-PCR results (Supplementary Fig. S1c). Skeletal muscles were harvested from mice of different ages and used for western blot analysis. KDM2B was highly expressed at embryonic day 18 (E18), and its expression was maintained for 1 month after birth, after which its expression gradually decreased with age (Supplementary Fig. S1d).

### KDM2B inhibits skeletal muscle cell differentiation

**Fig. 1:**
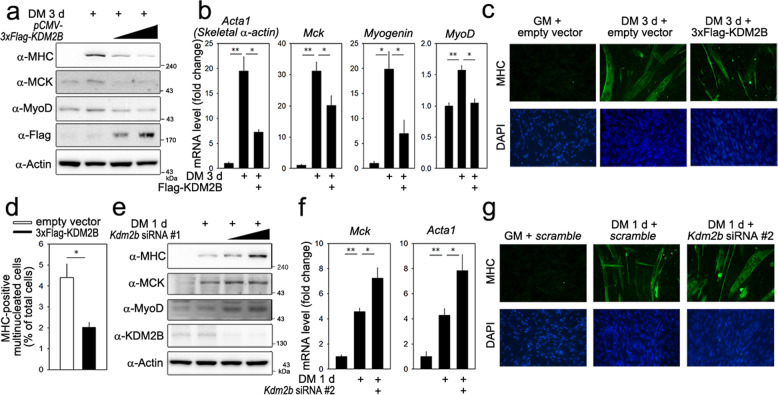
KDM2B inhibits myoblast differentiation. **a** Forced expression of KDM2B decreased the expression levels of skeletal muscle genes in C2C12 cells. Treatment with DM for 3 days induced the expression of myosin heavy chain (MHC), MyoD, and muscle creatinine kinase (MCK). These increases were attenuated when KDM2B was overexpressed. The overexpression of KDM2B was performed through the transfection of *pCMV-3xFlag-KDM2B*. The empty vector *pCMV-3xFlag* served as a mock control. **b** qRT-PCR analysis showing that the increased transcript levels of *skeletal α-actin (Acta1)*, *Mck*, *Myogenin*, and *MyoD* were downregulated by the overexpression of KDM2B. **c** Treatment with DM for 3 days induces elongation and multinucleation of C2C12 cells, which was attenuated by the forced expression of KDM2B. Immunocytochemistry was performed with an anti-MHC antibody. **d** Direct cell count after immunocytochemical analysis with MHC showing that MHC-positive multinucleated cell count was reduced when *KDM2B* was transfected. Images from 20–24 different fields were randomly obtained. Both MHC-positive and multinucleated cells, as well as total cells (stained with DAPI) were counted. **e** Knockdown of *Kdm2b* with *Kdm2b* siRNA #1 enhanced skeletal muscle gene expression induced by treatment with DM for 1 day. **f** qRT-PCR results for *Mck* and *Acta1*. **g** Immunocytochemical analysis with an anti-MHC antibody showed that elongation and multinucleation were enhanced by transfection with *Kdm2b* siRNA. *p < 0.05; **p < 0.01.

We next examined the effect of KDM2B in C2C12 cells. Replacement of the growth medium (GM) with DM for 3 days caused an increase in the expression of skeletal proteins, such as myosin heavy chain (MHC) and muscle creatinine kinase (MCK). These increases were blunted when KDM2B was overexpressed (Fig. 1a). Under the same experimental conditions, the expression level of exogenous KDM2B induced by transfection of 3xFlag-KDM2B was much higher than that of endogenous KDM2B (Supplementary Fig. S1e). Transfection of KDM2B did not alter cell survival when the cells were cultured in DM (Supplementary Fig. S1f). mRNA transcript levels of skeletal α-actin (actin alpha 1 skeletal muscle, *Acta1*) and *Mck*, both of which are terminal differentiation markers in myogenesis, were downregulated in the cells transfected with *KDM2B* (Fig. 1b). The expression of MyoD, a key myogenic transcription factor^20,21^, was also significantly lowered by KDM2B (Fig. 1a, b 4th panel), as was that of *Myogenin* (Fig. 1b). Treatment with DM for 3 days induced the elongation (Fig. 1c) and multinucleation of C2C12 cells, which was attenuated by transfection with *KDM2B* (Fig. 1d).

We also examined the effect of knocking down *Kdm2b* using siRNA. Culturing in DM for 1 day induced the expression of MHC, which was enhanced by *Kdm2b* siRNA (Fig. 1e, *Kdm2b* siRNA #1). Increases in MCK and MyoD expression were also potentiated (Fig. 1e). To rule out an off-target effect, we designed another siRNA that targeted a different location (*Kdm2b* siRNA #2). This siRNA also significantly induced the expression of myogenic proteins (Supplementary Fig. S1g). The increases in the mRNA levels of *Mck* and *Acta1* were enhanced by *Kdm2b* siRNA (Fig. 1f). Immunocytochemical analysis further showed that *Kdm2b* siRNA increased the formation of skeletal myofibers in the C2C12 cells induced after 1 day of culture with DM (Fig. 1g).

### KDM2B affects the transcription of myogenic genes by detaching SRF from the *SRE*

**Fig. 2:**
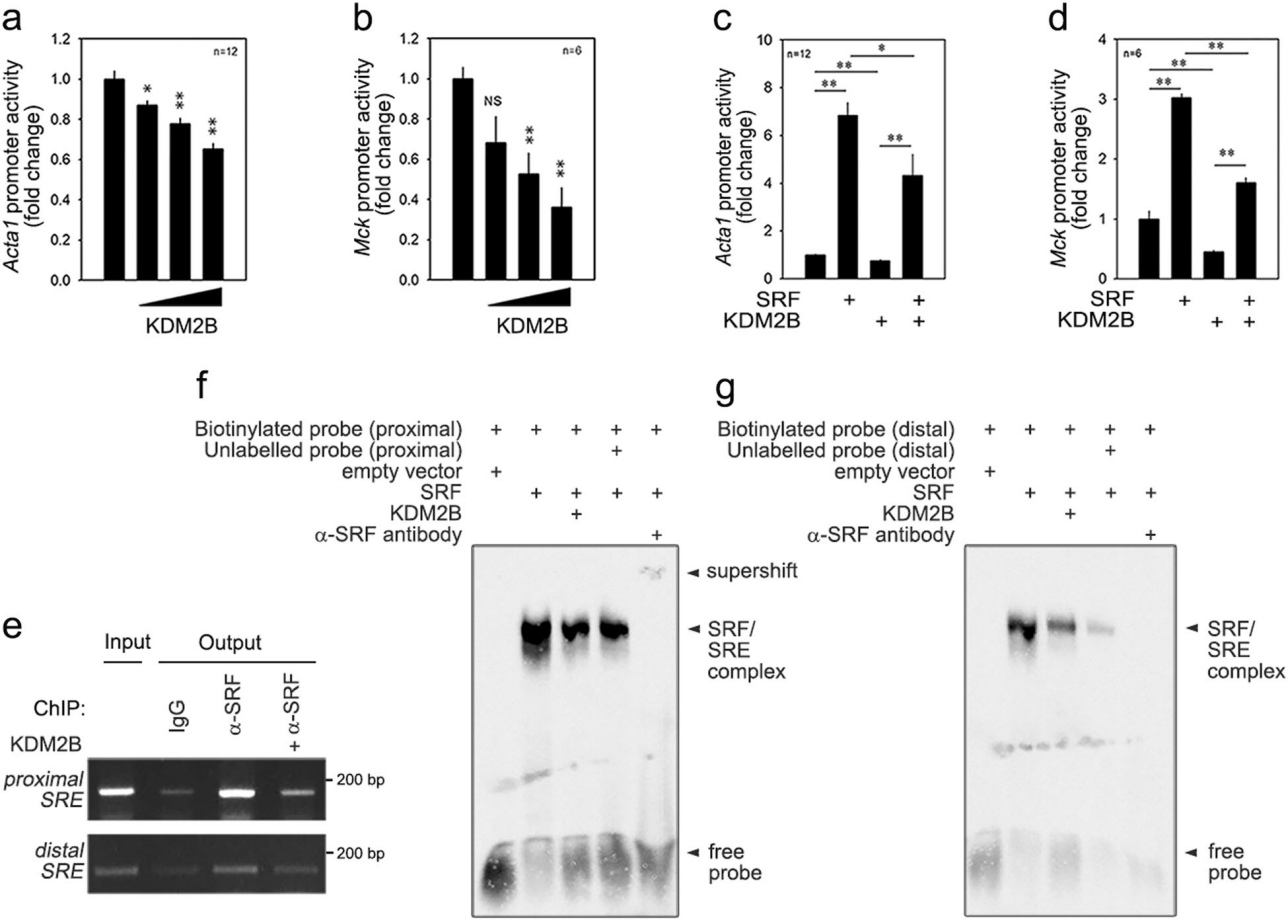
KDM2B transcriptionally inhibits serum response factor (SRF)-dependent muscle gene expression by detaching SRF from the *SRE*. **a**, **b** Transfection of *pCMV-3xFlax-KDM2B* (KDM2B) reduced the basal promoter activity of *Acta1* (**a**) and *Mck* (**b**) in a dose-dependent fashion. **c**, **d** SRF-induced transactivation of the *Acta1* (**c**) and *Mck* (**d**) promoters was significantly attenuated by cotransfection with *pCMV-3xFlax-KDM2B*. *pCGN-HA-SRF* was transfected to overexpress SRF. **e** Chromatin immunoprecipitation (ChIP) showing that KDM2B overexpression reduced SRF occupancy at the proximal or distal serum response element (*SRE*) of the *Acta1* promoter, indicating that KDM2B induced the detachment of SRF from the *SRE*. **f** Gel shift assay showing that the binding of SRF to the proximal *SRE* was attenuated by the addition of KDM2B. Nuclear extracts of the C2C12 cells transfected with *pCGN-HA-SRF* and/or *pCMV-3xFlag-KDM2B* were used. *pCGN-HA* or *pCMV-3x-Flag* empty vector was used as a mock control. **g** Gel shift assay with distal *SRE*. *p < 0.05; **p < 0.01; NS, not significant.

We next utilized promoter luciferase constructs and transfected the mammalian expression vector of KDM2B into C2C12 cells. The transfection of *Kdm2b* decreased the basal promoter activities of *Acta1* and *Mck* (Fig. 2a, b) in a dose-dependent fashion. *Kdm2b* also attenuated the basal promoter activity of *myogenin* (Supplementary Fig. S2a). We also tested whether KDM2B affected SRF-induced transactivation of skeletal myogenic genes^22,23^. We found that the transfection of *KDM2B* significantly attenuated the SRF-induced transactivation of the *Acta1* (Fig. 2c), *Mck* (Fig. 2d), and *Myogenin* (Supplementary Fig. S2b) promoters. As shown by chromatin immunoprecipitation (ChIP) analysis, binding of endogenous SRF to either the proximal or distal SRE in the *Acta1* promoter region was attenuated by the transfection of *KDM2B* in the C2C12 cells cultured in DM for 1 day (Fig. 2e and Supplementary Fig. S3a). Similarly, the binding of exogenous HA-tagged SRF to the *SRE* was reduced by the transfection of *KDM2B* in the C2C12 cells cultured in DM for 1 day (Supplementary Fig. S3b, c). In contrast, knocking down *Kdm2b* in C2C12 cells enhanced the binding of SRF to either the proximal or distal *SRE* in the *Acta1* promoter (Supplementary Fig. S3d).

We performed a gel shift assay with biotinylated *SRE* probes. Nuclear extracts from *SRF*-transfected C2C12 cells successfully retarded the gel mobility of the proximal *SRE* probe (2nd lane, Fig. 2f, arrowhead). However, the binding of SRF to the *SRE* was attenuated when nuclear extracts from the cells cotransfected with *KDM2B* and *SRF* were added (3rd lane). When the distal *SRE* probe was used, we observed the same result: KDM2B reduced the formation of the SRF-SRE complex (Fig. 2g).

### KDM2B inhibits transcription independent of histone methylation

Many researchers, including us, have demonstrated that KDM2B primarily targets histones to demethylate lysine residues^16,17,24^ and that histones H3K4me3 and H3K36me2, in particular, can be effectively demethylated^14,15^. These findings raise the possibility that our results showing the inhibition of myogenic transcription factors may be the result of demethylation at H3K4 and H3K36. Thus, using ChIP analysis, we determined the methylation status of the histones associated with the *SRE* in the promoter of *Acta1*. The histone H3K4me3 level associated with the proximal *SRE* in the *Acta1* promoter was not altered by the transfection of KDM2B in the C2C12 cells exposed to DM for 1 day. Similarly, KDM2B also did not affect the binding of H3K4me3 to the distal *SRE* (Supplementary Fig. S4a, b). H3K36me2 associated with either the proximal or the distal *SRE* was not significantly altered by KDM2B overexpression (Supplementary Fig. S4c, d). We also found that knocking down *Kdm2b* with siRNA did not increase the ‘active’ methylation of histones H3K4me3 (Supplementary Fig. S4e, f) or H3K36me2 (Supplementary Fig. S4g, h) at the two *SRE*s of *Acta1* in C2C12 cells in the GM condition.

### KDM2B directly binds to myogenic transcription factors

**Fig. 3:**
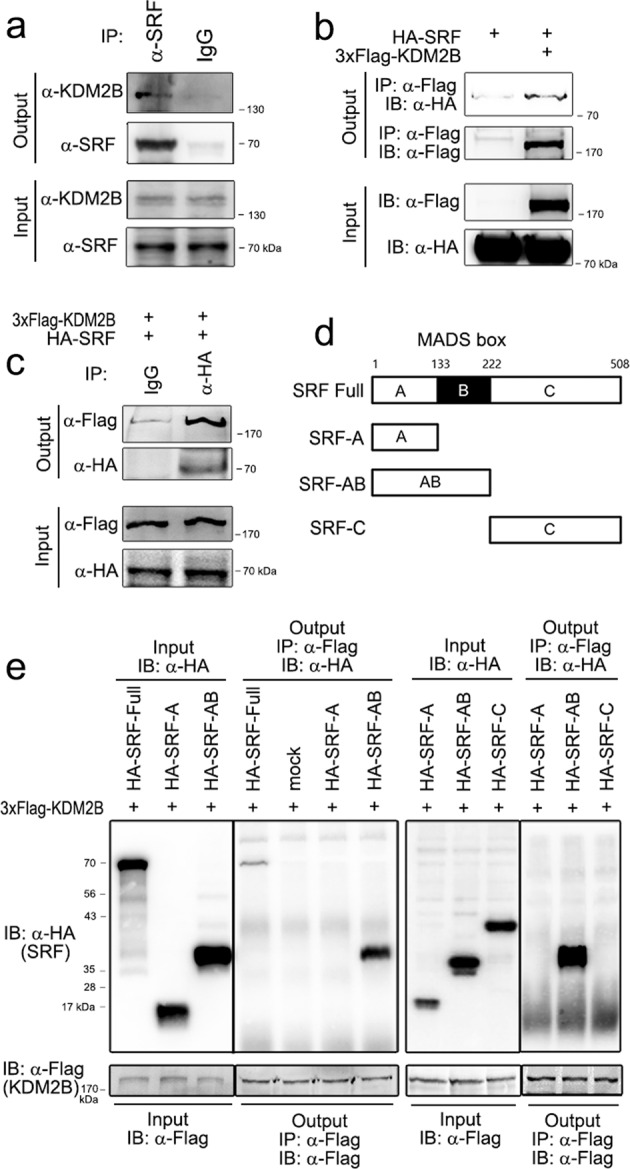
KDM2B directly interacts with SRF through its binding to the MADS box. **a** Endogenous SRF successfully recruited endogenous KDM2B in undifferentiated C2C12 cells. **b** Flag-tagged KDM2B physically bound to HA-tagged SRF. *pCMV-3xFlag-KDM2B* or *pCGN-HA-SRF* was transfected into 293T cells, and immunoprecipitation analysis was performed. **c** Inverse immunoprecipitation. HA-tagged SRF pulled down Flag-tagged KDM2B in 293T cells. **d** Structure of truncated SRF mutant proteins. The MADS-box consists of the 133–222 amino acid region (black boxed region). **e** The MADS-box interacted with KDM2B. Two left gels: SRF-full length, SRF-A, and SRF-AB constructs were used for domain-mapping analysis. Immunoprecipitation was performed with an anti-Flag (KDM2B) antibody. Two right gels: SRF-A, SRF-AB, and SRF-C constructs were used for immunoprecipitation. Notably, only full-length SRF and SRF-AB were recruited by KDM2B.

Many epigenetic regulators can induce the modifications of histones and other proteins, including transcription factors. Thus, we were interested in knowing whether KDM2B directly affects the posttranslational modification of key transcription factors in myogenesis. SRF is critical to the early phase of skeletal muscle cell differentiation because it is required for the transcriptional activation of muscle-specific genes^9^ that contain a well-conserved CArG box-binding motif [CC(A/T)6GG] in their promoter region^25^. Thus, we investigated whether KDM2B affects the PTM of SRF and thereby its transcriptional activity. In C2C12 myoblasts, KDM2B physically interacted with SRF (Fig. 3a–c).

We performed domain mapping analysis by using truncated mutant SRF proteins, as shown in Fig. 3d. We first examined the expression of SRF-A and SRF-AB truncation mutants (leftmost panel in Fig. 3e). An interaction with KDM2B was observed for SRF-AB (4th lane in the second panel in Fig. 3e), as well as full-length SRF (1st lane in the second panel), whereas an interaction with KDM2B was not observed for SRF-A (3rd lane in second panel). We also examined whether SRF-C interacts with KDM2B. All the truncation mutants (SRF-A, SRF-AB, and SRF-C) were adequately expressed (3rd panel from the left in Fig. 3e); however, only SRF-AB interacted with KDM2B (2^nd^ lane in the 4th panel from the left in Fig. 3e).

### The SRF MADS-box domain is methylated during myocyte differentiation

**Fig. 4:**
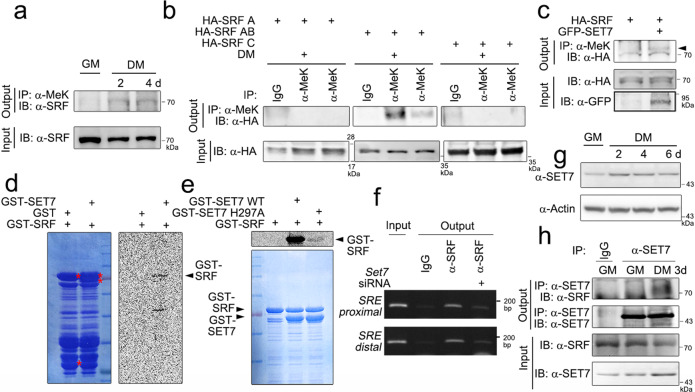
Myoblast differentiation induces SET7-dependent methylation of the SRF MADS-box. **a** Immunoprecipitation-based in vivo cellular methylation assay using anti-methylated lysine (anti-α-MeK) antibody revealed that SRF was methylated during C2C12 differentiation. Notably, methylation was increased in the cells cultured in differentiation medium (DM). **b** HA-tagged truncated mutants of *SRF* (*SRF-A, SRF-AB*, and *SRF-C*) were transfected into C2C12 cells, and then, the cells were subjected to serum deprivation (DM). The SRF MADS-box region was methylated, as determined with a α-MeK immunoprecipitation-based assay. **c** Transfection of *SET7* induced the methylation of SRF in the C2C12 cells. **d** In vitro methyltransferase assay in complete cell-free conditions showing that the chimeric protein GST-SET7 induced the methylation of GST-SRF. GST protein served as a control. Left: Coomassie blue staining was used to determine the expression of GST proteins. Right: Autoradiograph. **e** Enzymatically dead SET7 (SET7 H297A) failed to induce the methylation of SRF. **f** *Set7* siRNA attenuated the binding of SRF to the proximal *SRE* in the *Acta1* gene promoter. ChIP analysis. **g** Expression of SET7 was increased during C2C12 differentiation. **h** Association between endogenous SET7 and SRF was enhanced in DM.

What is the function of KDM2B in skeletal muscle differentiation? Is the methylation of muscle-specific transcription factors involved? To answer these questions, we first sought to determine whether myogenic transcription factors are methylated or demethylated during the differentiation of myoblasts, and we used an immunoprecipitation-based methylation analysis for this purpose. C2C12 cellular lysates were obtained from either the GM or DM (cultured for 2 and 4 days) and subjected to immunoprecipitation with anti-methyl-lysine antibody, and the methylated SRF was measured by immunoblot analysis with anti-SRF antibody. The SRF from the DM lysate showed increased the methylation levels (Fig. 4a). Next, we wanted to determine which SRF domain is critical for this methylation. To address this, we used truncated SRF mutants. Only SRF-AB was methylated in the DM condition (Fig. 4b).

The same group reporting that SET7 is required for skeletal myogenesis also suggested that SET7-induced methylation of histone H3K9 is required for normal skeletal muscle development^26^. Judson et al. reported that SET7 modulates the methylation status of β-catenin and promotes myogenic gene expression^26^. We also examined whether SET7 can methylate SRF. The transfection of *SET7* increased the methylation of SRF, as determined by immunoprecipitation with an anti-methylated lysine antibody (Fig. 4c). This finding was further supported by the results of an in vitro methylation assay. In contrast to the GST-only condition, a negative control, GST-SET7 induced the methylation of GST-SRF (Fig. 4d). SRF methylation was abolished when an enzymatically dead SET7 mutant (SET7 H297A)^27^ was used (Fig. 4e). The ChIP analysis clearly showed that the binding of SRF to the *SRE* is SET7-dependent, and the transfection of *Set7* siRNA reduced the proximal *SRE* occupancy of SRF in the *Acta1* promoter (Fig. 4f and Supplementary Fig. S5a).

The expression of SET7 was increased in the acute phase of myocyte differentiation after exposure to DM for 2 and 4 days (Fig. 4g), a finding supported by the examination of the mRNA levels (Supplementary Fig. S5b). The association between SET7 and SRF was enhanced in the DM condition compared with the GM condition (Fig. 4h) because of the increase in the amount of SET7 and SRF in the DM condition (lower panel input). The transfection of *SET7* further increased SRF-induced transactivation of *Acta1* (Supplementary Fig. S5c).

### SRF methylation of lysine residues is induced by SET7 or differentiation medium

**Fig. 5:**
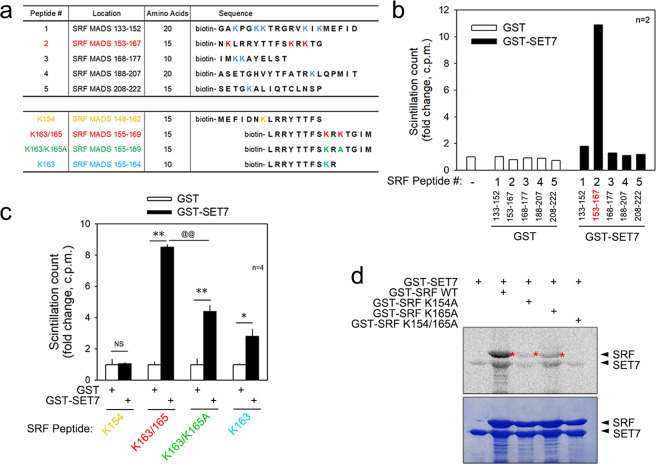
SRF lysine residues are methylated by SET7. **a** Synthetic peptides were used in this study to determine methylated lysine residues. **b** Only peptide #2, spanning amino acids 153–167 in the MADS-box of SRF, was methylated by GST-SET7. SRF synthetic peptides were used as substrates and incubated with GST-SET7 in the in vitro methyltransferase assay. **c** To determine the critical lysine, K154-, K165-, and K165A-bearing SRF synthetic peptides were generated and used in the in vitro methyltransferase assay. SRF K154 did not show any methylation, whereas SRF K165 emitted a strong methylation signal. Notably, SRF K165A showed one-half the methylation as the WT peptide, which suggested methylation of other lysine residues in addition to SRF K165. **d** GST-SRF WT, GST-SRF K154A, GST-SRF K165A, and GST-SRF K154/165A were used in the in vitro methyltransferase assay. For the methylation enzyme, GST-SET7 was supplemented. Notably, the methylation was nearly completely abolished when both K154 and K165 were substituted with alanine, which suggests that both SRF K154 and K165 are methylated by SET7. *p < 0.05; ** and ^@@^*p* < 0.01; NS, not significant.

To investigate the biological role of SRF methylation, we needed to determine which lysine residue is critical for SET7-mediated methylation. Thus, as shown in the upper table in Fig. 5a, we generated 5 synthetic peptides spanning the whole MADS-box in SRF and performed an in vitro methyltransferase assay. When GST-SET7 was coincubated with these peptides, SRF peptide #2, spanning amino acids 153~167 and containing K154, K163, and K165, was highly methylated (Fig. 5b). However, SET7 H297A, an enzymatically dead SET7 mutant, failed to induce the methylation of peptide #2 (Supplementary Fig. S5d).

We next generated two SRF peptides: K154 and K163/165. K154 spans amino acids 148–162, whereas K163/165 contains amino acids 155–169 (Fig. 5a lower table). In contrast with the effect of K154 (1st group of bars in Fig. 5c), the methylation of the peptide spanning K163/165 was significantly increased (2nd group of bars in Fig. 5c). The SRF K163/165 peptide spanning 155–169 contains two lysine residues. To specify which lysine residue is methylated, we first generated an SRF K165-dead mutant by substituting K165 with alanine: K163/K165A (Fig. 5a lower table). Interestingly, the methylation of the K163/K165A peptide was significantly lower than that of the K163/165 peptide (3rd group of bars in Fig. 5c). Therefore, we generated the SRF K163 peptide, which spanned 155–164, where only one lysine, K163, was included. K163 was sufficiently methylated by the addition of GST-SET7 (4th group of bars in Fig. 5c).

We also performed an in vitro methyltransferase assay using GST-SRF fusion proteins. GST-SET7 successfully induced the methylation of wild-type (WT) GST-SRF under in vitro conditions (the 2nd lane of the upper gel in Fig. 5d and the 2nd lane of the right gel in Supplementary Fig. S5e). We also generated the following GST proteins: SRF K154A, K165A, and K154/165A. The substitution of K154 with alanine abolished the methylation of SRF (3rd lane in Fig. 5d). The methylation level was also significantly reduced in the GST-SRF K165A peptide (4th lane in Fig. 5d and 3rd lane in Supplementary Fig. S5e) and in the GST-SRF K154/165A peptide (5th lane in Fig. 5d). These in vitro methylation results suggest that SET7 might induce the methylation of SRF at K154, K163, and K165.

### The methylation of K165 is critical for SRF transactivation and muscle cell differentiation

**Fig. 6:**
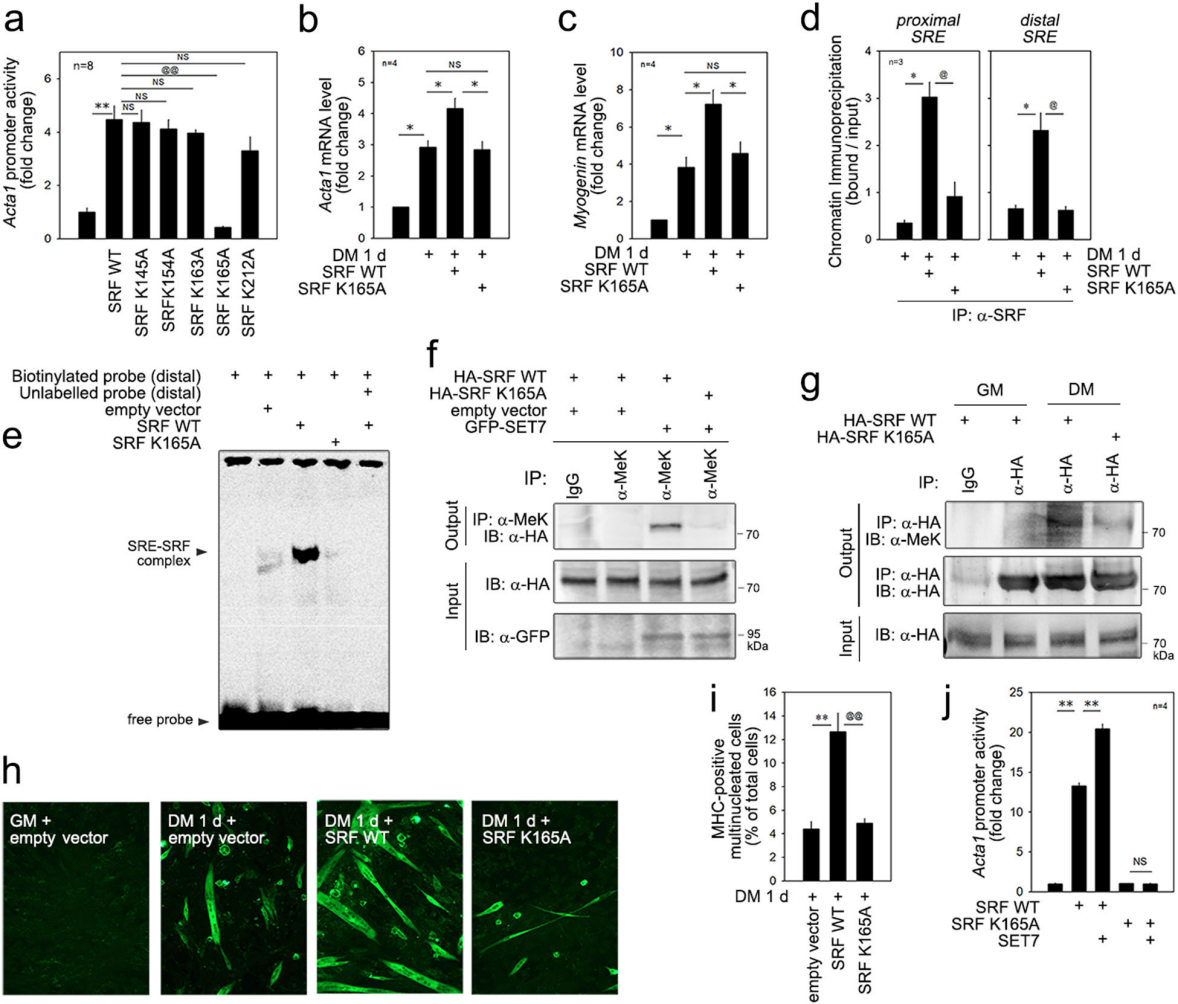
SRF K165 is a biologically active methylation site involved in muscle differentiation. **a** Promoter analysis using the *Acta1* promoter revealed that, among the peptides tested, only SRF K165A lost its basal transcriptional activity. Notably, SRF K154A retained sufficient promoter activity, which was comparable to that of the wild-type peptide. **b** DM induces *Acta1* mRNA expression, which was further potentiated by the transfection with *SRF WT*. However, this enhancement was not observed when *SRF K165A* was transfected. **c** *Myogenin* mRNA level. **d** ChIP analysis showing that SRF K165A loses its ability to bind to two *SRE*s (CArG-proximal and CArG-distal) in the *Acta1* promoter. To pull down either SRF WT or SRF K165A, an anti-HA antibody was used in the ChIP analysis. **e** Gel shift assay showing that SRF K165A loses its binding affinity for the distal *SRE*. **f** Immunoprecipitation-based in vivo cellular methylation assay showing that the transfection of *SET7* induced the methylation of SRF WT, whereas it failed to methylate SRF K165A. **g** DM induced the methylation of SRF WT, whereas it failed to methylate SRF K165A. **h** Elongation and tube formation induced by treatment with DM for 1 day was enhanced by transfection with *pCGN-HA-SRF WT* (SRF WT), whereas this enhancement is not seen with transfection with *pCGN-HA-SRF K165A* (SRF K165A). Immunocytochemical analysis with anti-MHC antibody. **i** MHC-positive multinucleated cell count. **j** Transfection of *SET7* enhanced SRF WT-induced transactivation of the *Acta1* promoter but failed to activate SRF K165A. * and ^@^*p* < 0.05; ** and ^@@^*p* < 0.01; NS, not significant.

In previous studies, we found that SET7 induces the methylation of the MADS box domain of SRF and that K154, K163, and/or K165 might be SET7-dependent methylation targets. Notably, however, that not all the methylated lysine residues were biologically functional. Thus, we tried to narrow down the specific methylation residues that are biologically active. To accomplish this aim, we generated diverse mutants by site-directed mutagenesis in which the lysine residues were replaced by alanine residues (Supplementary Fig. S6a). The SRF-induced transactivation of the *Acta1* promoter was completely abolished when *SRF K165A* was transfected (Fig. 6a). Interestingly, transactivation was not altered when other lysine residues in the MADS-box were mutated, which suggests that K165 is the key lysine. In addition to basal *Acta1* promoter activity, SRF K165A failed to further increase *Acta1* promoter activity (Fig. 6b). The result was similar when a *myogenin-luciferase* construct was used (Fig. 6c). The ChIP analysis showed that *SRE* occupancy by SRF in the *Acta1* promoter was reduced when *SRF K165A* was transfected (Fig. 6d). A representative gel is shown in Supplementary Fig. S6b. Using a gel shift assay, we further studied whether SRF K165 methylation affected the binding of SRF to the distal *SRE* (Fig. 6e). The nuclear extract of *SRF K165A*-transfected C2C12 cells failed to form an SRF-SRE complex (4th lane), in contrast to the *SRF WT*-transfected cells (3rd lane). Similarly, complex formation between SRF K165A and the proximal *SRE* was significantly reduced (Supplementary Fig. S6c).

The methylation of SRF was examined by in vivo cellular studies; transfection of *SET7* induced the methylation of SRF WT (the 3rd lane of the upper gel in Fig. 6f). SET7 failed to induce methylation in SRF K165A (4th lane). We next examined whether SRF was methylated in DM in the absence of forced expression of SET7. Treatment of C2C12 cells with DM increased the methylation of SRF WT (the 2nd vs. the 3^rd^ lane of the upper gel of Fig. 6g). However, the DM did not induce the methylation of SRF K165A (4th lane).

The myogenic activity of SRF K165A was examined. Treatment of C2C12 myoblasts with DM for 1 day induced multinucleation and elongation, which were then enhanced by transfection of *wild-type SRF*. However, SRF K165A failed to enhance cell multinucleation and elongation (Fig. 6h). The quantification of the multinucleated cells further showed that the substitution of SRF K165 with alanine abolished the SRF-induced increase in multinucleation (Fig. 6i). The transfection of *SET7* further increased the SRF-mediated transactivation of the *Acta1* promoter (2nd vs. 3rd lane in Fig. 6j). However, no further activation was observed when *SRF K165A* was transfected (4th vs. 5th lane).

We tested whether the substitution of K165 with alanine attenuated the binding of SRF to either MyoD or SET7. In our experimental model, the association of these proteins was not altered (Supplementary Fig. S6d, e).

### KDM2B demethylates K165 of SRF

**Fig. 7:**
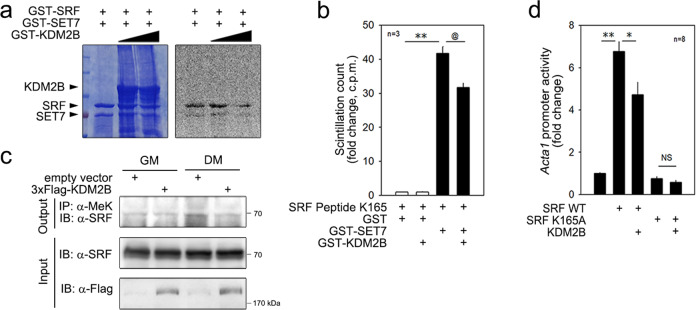
KDM2B demethylates SRF WT to mimic SRF K165A. **a** GST-KDM2B reduced the methylation of SRF in a dose-dependent fashion as indicated by an in vitro methylase assay. **b** In vitro methylase assay with SRF synthetic peptide. GST-SET7 induced the methylation of the SRF K165 peptide, an effect partially attenuated by the addition of GST-KDM2B. **c** Immunoprecipitation-based in vivo methylation assay. The DM-induced methylation of SRF was reduced by the transfection of *KDM2B* in C2C12 cells. **d** Transfection with *pCMV-3x-Flag-KDM2B* reduced the *pCGN-HA-SRF WT*-induced transactivation of the *Acta1* promoter, whereas it failed to affect *pCGN-HA-SRF K165A*. * and ^@^*p* < 0.05; **p < 0.01; NS, not significant.

Next, we checked whether KDM2B demethylates SRF. The in vitro methylation assay showed that the addition of GST-KDM2B attenuated the GST-SET-induced methylation of GST-SRF (Supplementary Fig. S7a) in a dose-dependent fashion (Fig. 7a). The in vitro methylation assay with synthetic peptide showed that KDM2B demethylates SRF K165; KDM2B reduced SET7-induced methylation when the K165 peptide (Fig. 7b) or peptide #2 spanning 153–167 amino acids of SRF (Supplementary Fig. S7b) was used as the substrate. In contrast, with the intact K165 peptide, whose methylation was significantly reduced by KDM2B (1st vs. 2nd bar in Supplementary Fig. S7c), no further reduction in methylation was observed when peptide K165A was used as a substrate (3rd vs. 4th bar in Supplementary Fig. S7c). Treatment with DM increased the methylation of endogenous SRF in C2C12 cells, as determined by an in vivo immunoprecipitation-based methylation assay (3rd lane in Fig. 7c). The increase in methylation was blunted when *KDM2B* was transfected into C2C12 cells (4th lane in Fig. 7c).

We next questioned whether the KDM2B-mediated removal of SET7-induced methylation resulted in transcriptional regulation under in vivo cellular conditions. As shown in Fig. 6i, SET7 further enhanced SRF-induced transactivation of the *Acta1* promoter. This enhancement, however, was abolished by cotransfection of *KDM2B* in a dose-dependent fashion (Supplementary Fig. S7d).

To learn whether SRF K165A mimics demethylate SRF, we first assumed that KDM2B does not further inhibit the transcriptional activity of SRF K165A when K165 is the only target of KDM2B for transcriptional regulation. As shown in Supplementary Fig. S2a, b, SRF WT-induced transactivation of the *Acta1* promoter was blunted by the transfection of *KDM2B* in C2C12 cells (2nd and 3rd bars, Fig. 7d). SRF K165A failed to transactivate the *Acta1* promoter, as shown in Fig. 6a (5th bar). Importantly, *KDM2B* transfection did not further reduce SRF K165A activity, in contrast to its effect on SRF WT activity (4th and 5th bar, Fig. 7d).

### SET7 inhibitors mimic KDM2B

**Fig. 8:**
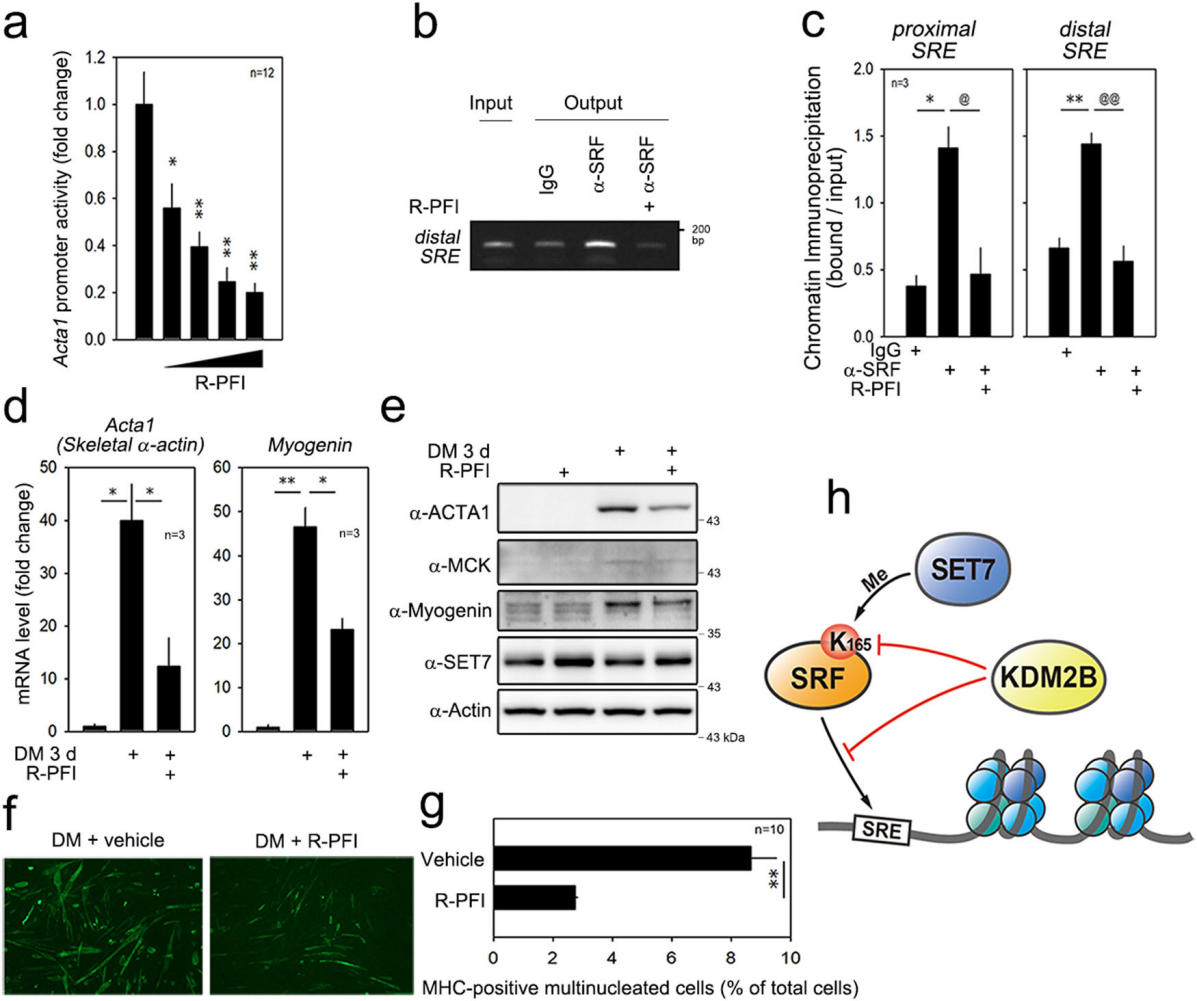
R-PFI, a SET7 inhibitor, attenuates myoblast differentiation by the transcriptional repression of SRF-dependent genes. **a** R-PFI inhibited *Acta1* promoter activity in a dose-dependent fashion. **b** Representative gel showing the ChIP assay results. R-PFI attenuated the binding of SRF to the distal *SRE* in the *Acta1* gene promoter. **c** Quantification results of the ChIP analysis. **d** Quantitative RT-PCR results showing that R-PFI abolished the increases in *Acta1* and *Myogenin* mRNAs induced by DM for 3 days. **e** R-PFI blocked the increases in the amounts of ACTA1, MCK, and Myogenin induced by DM for 3 days. **f** Immunofluorescence images of α-MHC. **g** Multinucleated cell count. * and ^@^*p* < 0.05; ** and ^@@^*p* < 0.01. **h** Working hypothesis: KDM2B inhibits skeletal muscle differentiation by inhibiting SRF-induced transactivation of skeletal muscle genes. KDM2B directly binds to SRF to demethylate K165, which results in the detachment of SRF from the *SRE* in skeletal muscle-specific genes. SET7 counteracts KDM2B by methylating SRF K165.

The methyltransferase activity of SET7 is known to be inhibited by (R)-8-fluoro-N-(1-oxo-1-(pyrrolidin-1-yl)-3-(3-(trifluoromethyl)phenyl)propan-2-yl)-1,2,3,4-tetrahydroisoquinoline-6-sulfonamide hydrochloride [(R)-PFI-2, or R-PFI]^28^. Treatment with sinefungin (Sine), a pan inhibitor of methyltransferases^10^, did not affect C2C12 cell survival. However, R-PFI induced cell proliferation (Supplementary Fig. S8a). R-PFI inhibited the activity of the *Acta1* promoter in a dose-dependent manner (Fig. 8a). Treatment of the C2C12 cells with R-PFI attenuated the binding of SRF to the proximal and the distal *SRE* (Fig. 8b, c). The R-PFI-induced reduction in SRF-dependent transactivation resulted in decreased expression of the mRNAs of myogenic genes such as *Acta1* and *myogenin* (Fig. 8d) and their protein products (Fig. 8e). Indeed, R-PFI reduced the expression of MHC (Fig. 8f) and the number of multinucleated C2C12 cells (Fig. 8g).

Sine also inhibited *Acta1* promoter activity in a dose-dependent manner (Supplementary Fig. S8b) and the capacity of SRF to bind to the proximal and the distal *SRE* (Supplementary Fig. S8c, d). The mRNA levels and subsequent protein levels of both *Acta1* and *Myogenin* were reduced by treatment with Sine (Supplementary Fig. S9a, b). Sine also inhibited the expression of MHC proteins (Supplementary Fig. S9c) and led to a decrease in multinucleated cells (Supplementary Fig. S9d).

## Discussion

In this work, we have shown a novel function of the methylation of SRF in myogenic differentiation. We observed that KDM2B inhibits myogenic differentiation by repressing SRF-dependent transcriptional activity. This transcriptional repression was not caused by the demethylation of histones. Instead, we found that SRF, a nonhistone target, is methylated during myogenic differentiation in a SET7-dependent fashion and that KDM2B-induced demethylation contributes to transcriptional repression by inducing the detachment of SRF from the *SRE* (Fig. 8h).

Epigenetic modifiers finely regulate muscle differentiation not only through histone modification but also by the PTM of muscle-specific transcription factors. For example, SET7 mediates histone H3K9 methylation^26^, whereas lysine methyltransferase G9a methylates the key myogenic transcription factor MyoD^29^. Moreover, G9a methylates K267 of MEF2D and represses its transcriptional activity, while LSD1 demethylates it to induce myogenic differentiation^30,31^. KDM4A, an alternate histone demethylase, is required for skeletal muscle differentiation^32^ and regulates myogenesis in C2C12 cells by interacting with MyoD^33^. In this study, we found that KDM2B inhibits myogenesis. Notably, KDM2B inhibits myogenic differentiation in a histone methylation-independent manner.

In this study, we clearly showed that SET7 functions as an SRF-methyltransferase. SET7-induced histone methylation has been emphasized in muscle biology^26^. SET7 induces monomethylation of a histone (H3K4me1), which results in the recruitment of SRF at the promoter. Here, we suggest that SET7 counteracts KDM2B in regulating the methylation of SRF K165 and thereby modulates the occupancy of the *SRE* for transcriptional activation. Our data provide unique insights into the role of the demethylation of SRF in the control of myogenic differentiation. Moreover, our data indicate that SRF methylation, without altering histone methylation, plays a significant role in the KDM2B-mediated inhibition of myogenesis.

## Supplementary information

Supplementary Data
