# Supplementary material for: SRF is a nonhistone methylation target of KDM2B and SET7 in the regulation of skeletal muscle differentiation
Source: Exp Mol Med. 2021 Feb 9;53(2):250–63. doi: 10.1038/s12276-021-00564-4 (PMC8080764; doi:10.1038/s12276-021-00564-4)
Supplement: Supplementary file 1 — Supplementary Data [file 12276_2021_564_MOESM1_ESM.docx]

**
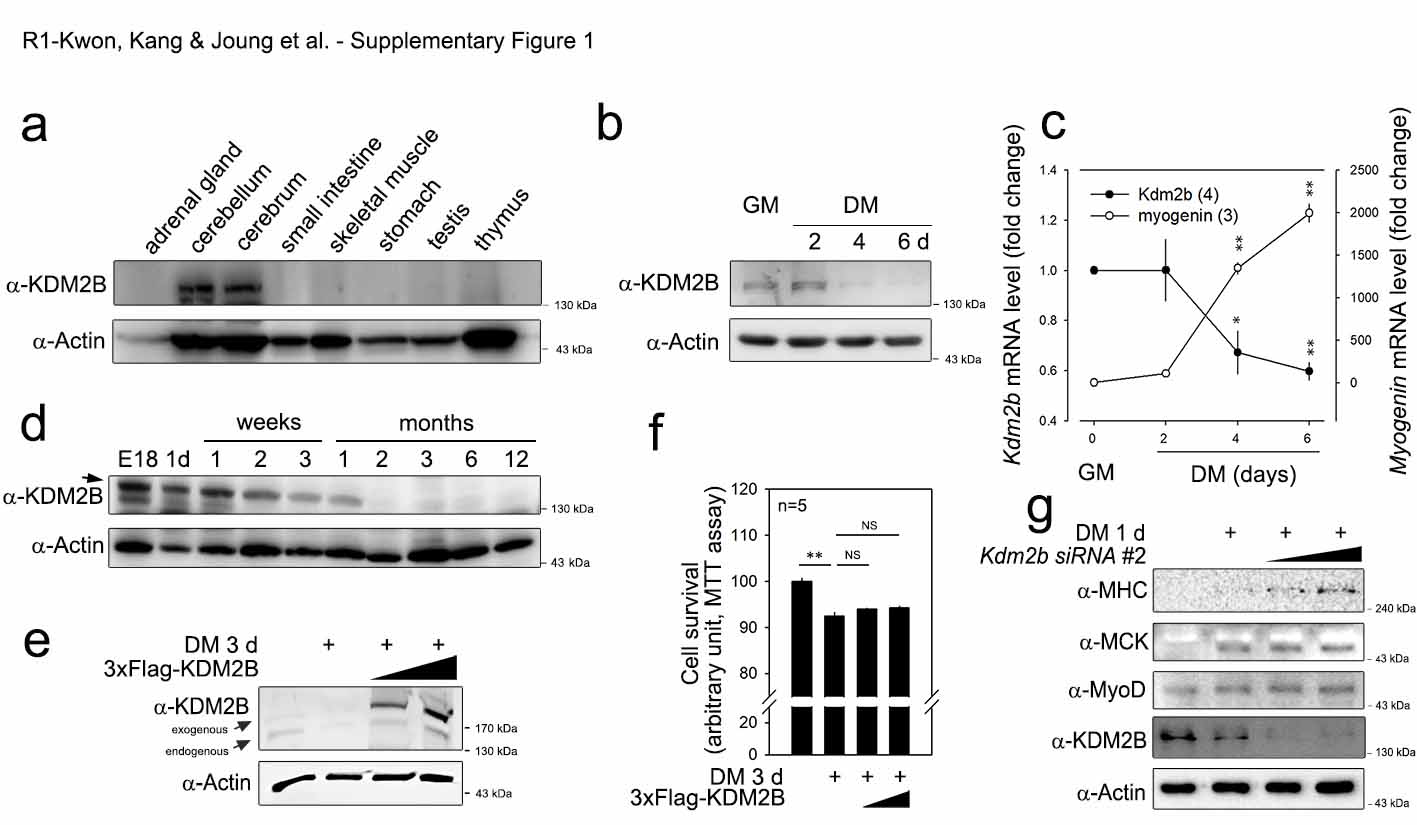
Supplementary information (SI)**

**Supplementary Fig S1. *Kdm2b* is expressed in the early phase of skeletal muscle development.** a. Tissue blot analysis showing the expression of KDM2B in adult mouse. b. KDM2B expression is gradually reduced in differentiation media (DM) in a C2C12 myoblast cell line. GM: growth media. c. Quantitative real-time PCR (qRT-PCR) showing that *Kdm2b* expression is down-regulated 4 and 6 days after treatment with DM in C2C12 cells. By contrast, *Myogenin*, a myogenic transcription factor, is increased during myoblast differentiation compared with mRNA levels in GM. Numbers in parentheses are the number of independent observations. d. Mouse tissue blot analysis showing that KDM2B is abundant in the early phase of skeletal muscle maturation. Skeletal muscle from mice at the ages indicated was used. Note that KDM2B is gradually down-regulated after birth. e. C2C12 cells were transfected with *pCMV-3xFlag-KDM2B* and both exogenous (upper bands) and endogenous (lower bands) KDM2B were probed with anti-KDM2B antibody. Flag-tagged exogenous KDM2B protein was 6 times as abundant compared with endogenous KDM2B. f. Transfection of KDM2B did not alter cell survival under the DM condition as determined with the MTT assay. g. Knock-down of *Kdm2b* with *Kdm2b* siRNA #2 enhances the skeletal muscle gene expression induced by treatment with DM for 1 d. To rule out the off-target effect, a different siRNA other than #1 (Fig 1g) was used. *p < 0.05; **p < 0.01

**
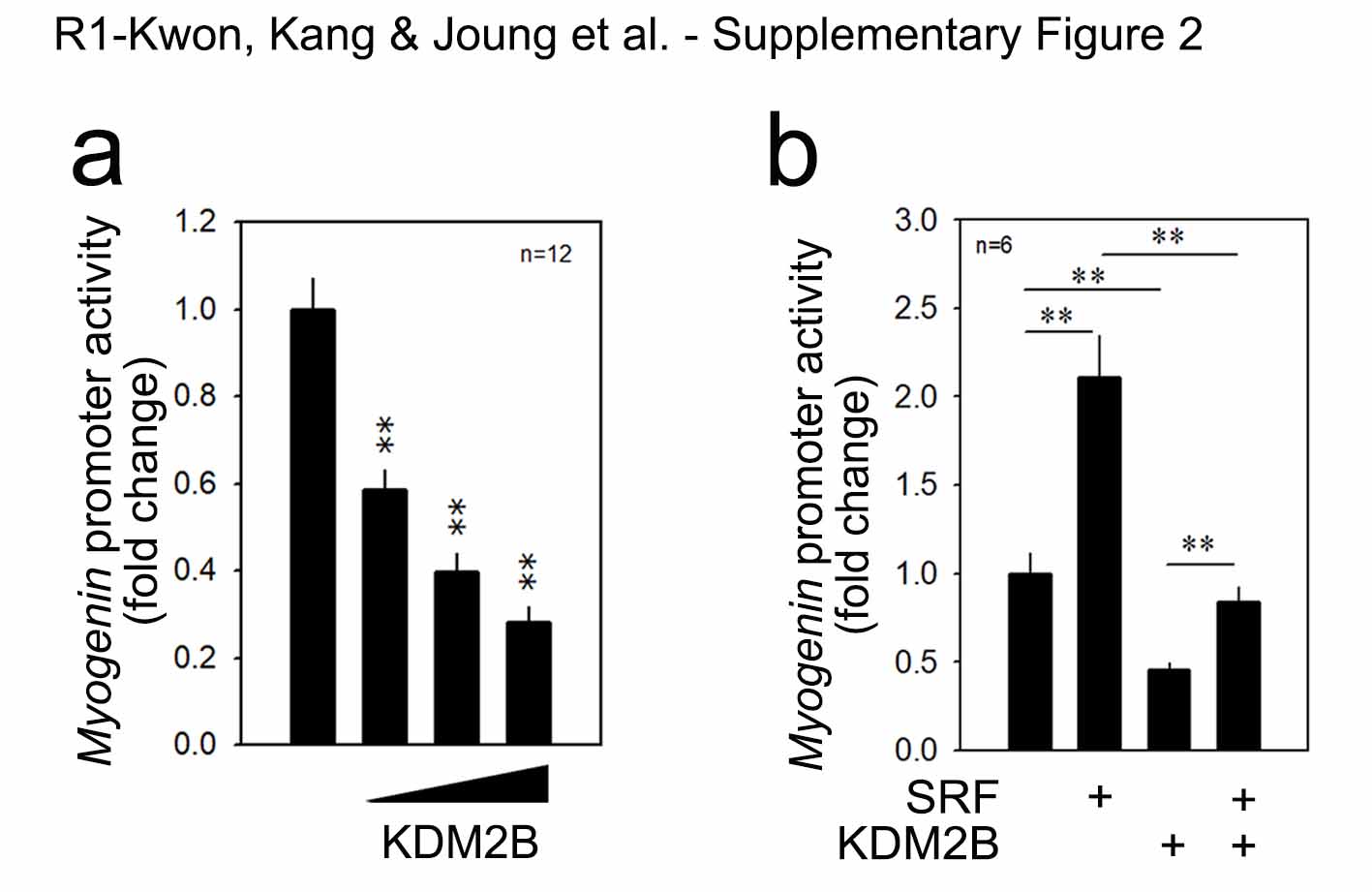
Supplementary Fig S2. SRF-dependent transactivation is attenuated by KDM2B.** a. Transient transfection of *pCMV-3xFlag-KDM2B* attenuates basal *Myogenin* promoter-luciferase activity in a dose-dependent fashion. b. Transactivation of *Myogenin* promoter induced by SRF is blocked by overexpression of KDM2B. **p < 0.01.

**
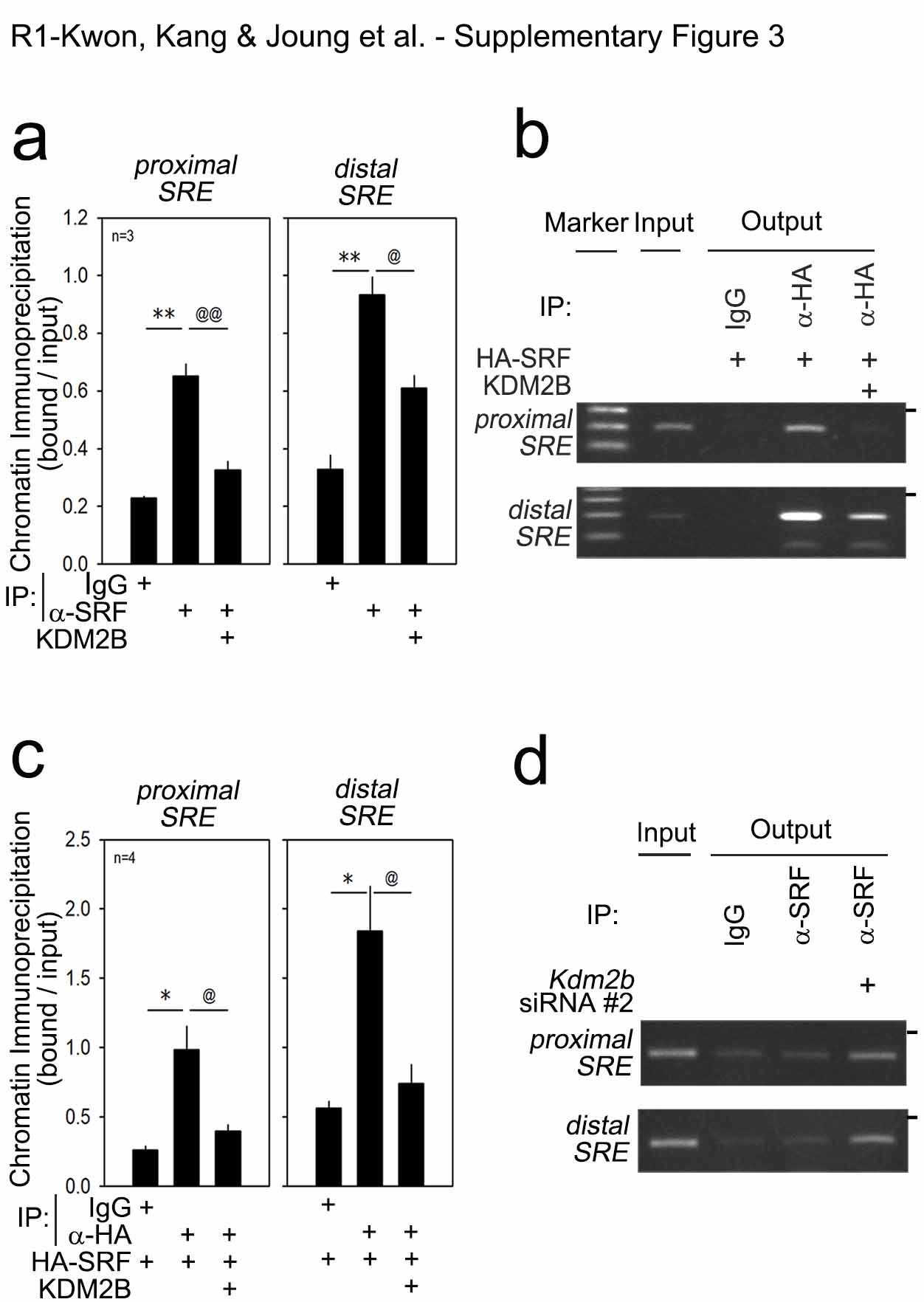
**

**Supplementary Fig S3. KDM2B induces detachment of SRF from the *SRE.*** a. Quantitative results of ChIP analysis. KDM2B detached endogenous SRF from either the proximal or the distal *SRE* on the *Acta1* promoter, which encodes skeletal α-actin. b,c. ChIP analysis. KDM2B detached exogenous HA-SRF. ChIP gel picture (b). Quantification results (c). d. Chip analysis showing the enhancement of SRF binding to the *SRE* by knocking-down of *Kdm2b* with siRNA #2. * and @ p < 0.05; ** and @@ p<0.01.

**
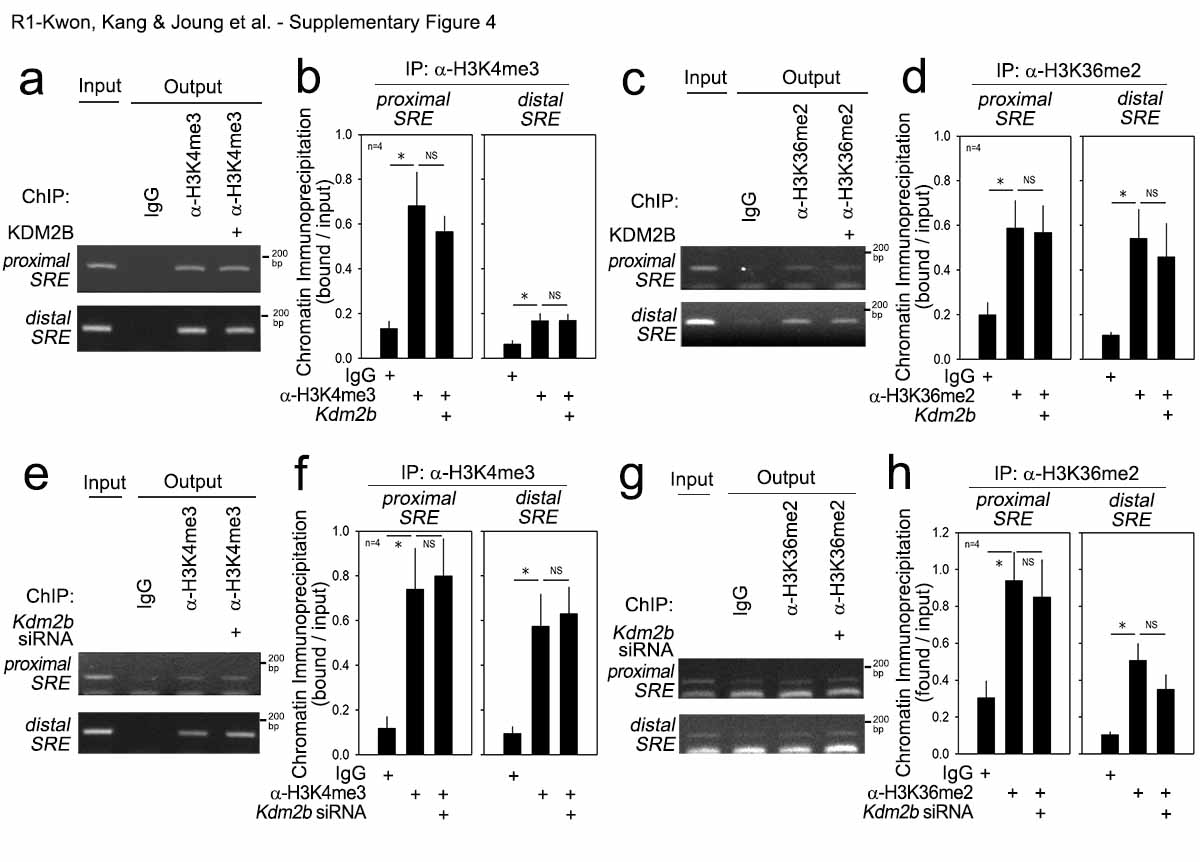
Supplementary Fig S4. KDM2B does not affect methylation of histone H3K4me3 and H3K36me3.** a,b. Treatment with DM for 1 d induces tri-methylation of histone H3K4 (H3K4me3) that is associated with either the proximal or distal *SRE* in the *Acta1* promoter. Transfection of *KDM2B* does not affect H3K4me3. Anti-H3K4me3 antibody was used for ChIP. (a) Representative gel picture. (b) Quantitative results. c,d. Results of di-methylation of histone H3K36 (H3K36me2) after KDM2B overexpression. (c) Representative gel picture. (d) Quantitative results. e,f. Results of tri-methylation of histone H3K4 (H3K4me3) after knocking-down of KDM2B. *Kdm2b* siRNA does not affect the H3K4me3 of the *SRE* in the *Acta1* gene promoter. (e) Representative gel picture. (f) Quantitative results. g,h. Results of di-methylation of histone H3K36me2 after knocking-down of *Kdm2b*. (g) Representative gel picture. (h) Quantitative results. C2C12 cells were used. * p < 0.05; NS, not significant.

**
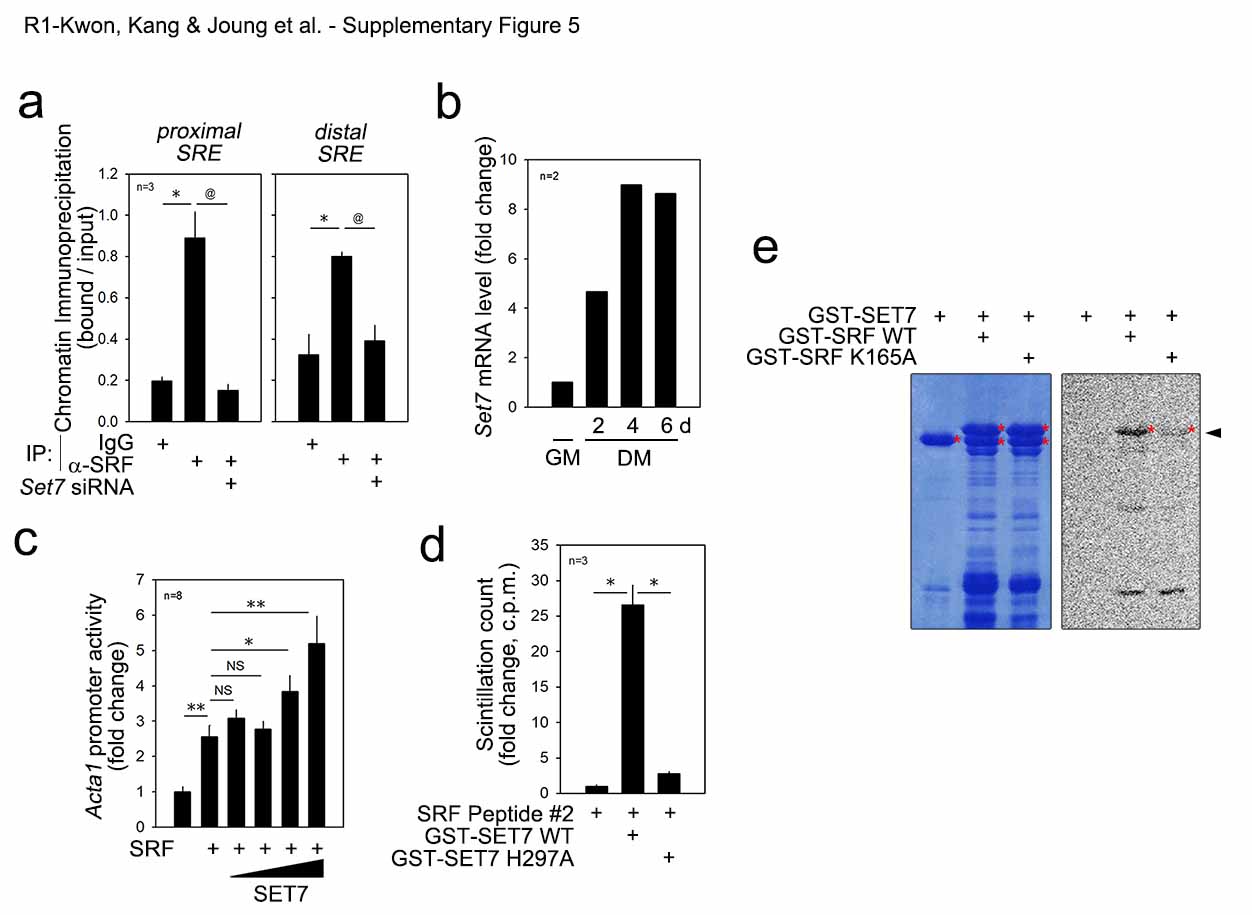
Supplementary Fig S5. SET7 physically interacts with SRF and induces its transcriptional activation.** a. *Set7* siRNA reduced binding of SRF to the *SRE* in the *Acta1* gene promoter. Quantification results of ChIP analysis. b. *Set7* mRNA level is dramatically increased during skeletal myoblast differentiation as shown by qRT-PCR analysis. c. Promoter analysis showing that SRF-induced transactivation of *Acta1* is further potentiated by co-transfection of *SET7*. d. Inert SET7 mutant (SET7 H297A) fails to induce methylation of SRF synthetic peptide #2 (153-167), which potentiates methylation by *SET7* WT. e. *in vitro* methylase assay. GST-SET7 induces methylation of GST-SRF WT but not that of GST-SRF K165A. * and @ p < 0.05; ** p<0.01; NS, not significant.

**
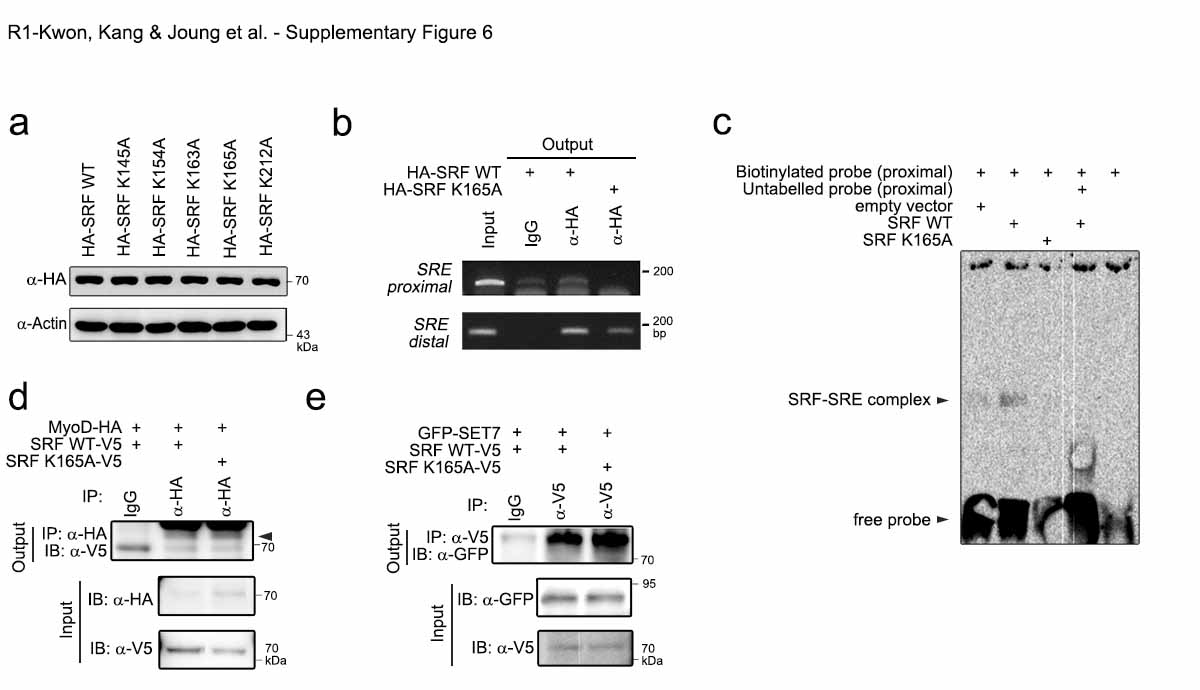
 Supplementary Fig S6. SRF K165A loses its binding ability to the *SRE*.** a. Expression of SRF mutant proteins. b. ChIP analysis. SRF K165A fails to bind to *SRE*. c. Gel shift assay showing that SRF K165A loses its binding capacity to the proximal *SRE*. d,e. Interaction of SRF with MyoD (d) and SET7 (e) is not altered in SRF K165A.

**
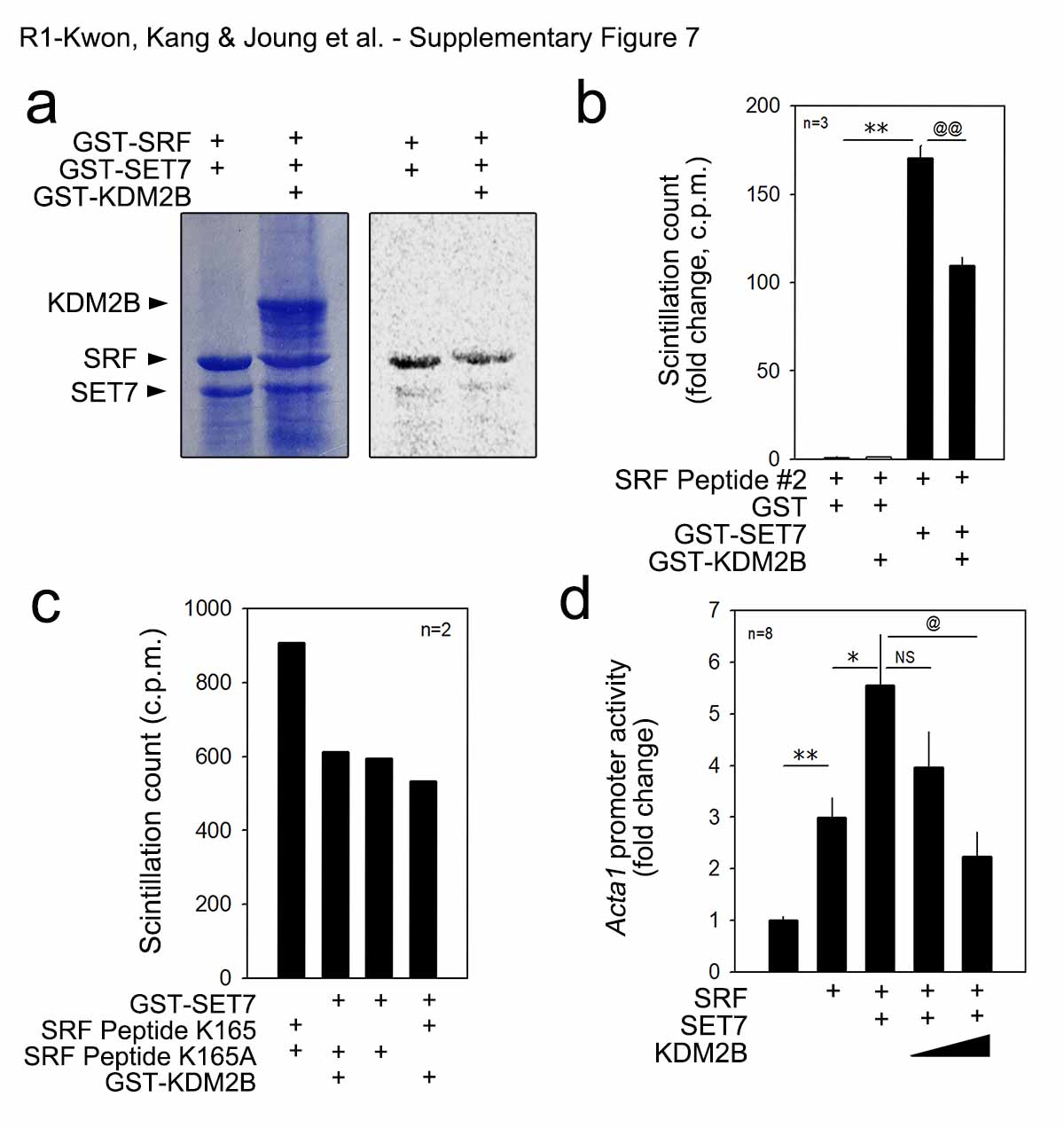
Supplementary Fig S7. KDM2B induces demethylation of SRF K165 and inhibits SET7-induced transactivation of the *Acta1* gene.** a. Autoradiograph image showing *in vitro* demethylase assay. GST-SRF methylation-induced by GST-SET7 is reduced by adding GST-KDM2B. b. GST-SET7-induced methylation of SRF synthetic peptide #2 spanning 153-167 is significantly attenuated by the addition of GST-KDM2B. c. GST-SET7-induced methylation of synthetic peptide flanking SRF K165 is attenuated by the addition of GST-KDM2B. However, GST-KDM2B fails to attenuate the methylation of a peptide flanking SRF K165A. d. SET7 enhanced SRF-induced transactivation of the *Acta1* promoter, which is then blocked by co-transfection with *KDM2B*. * and @, p < 0.05; ** and @@, p < 0.01; NS, not significant.

**
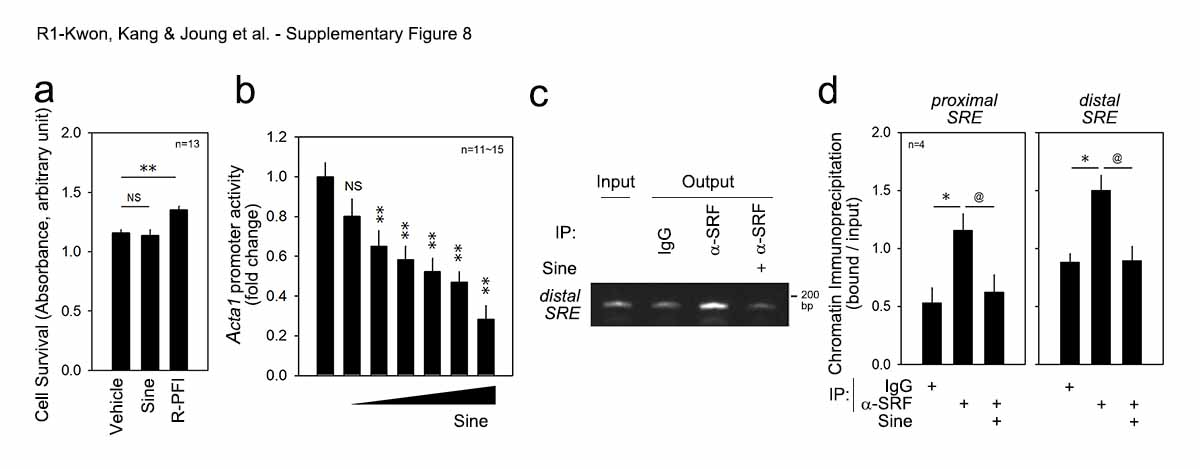
Supplementary Fig S8. Sinefungin (sine), an SET7 inhibitor, blocks transactivation of *Acta1* gene by detaching SRF from the *SRE*.** a. Cell survival measured by MTT assay. b. *Acta1* promoter analysis. c. Representative gel picture of ChIP. d. Quantitative results of ChIP analysis. * and @, p < 0.05; **p < 0.01; NS, not significant.

**
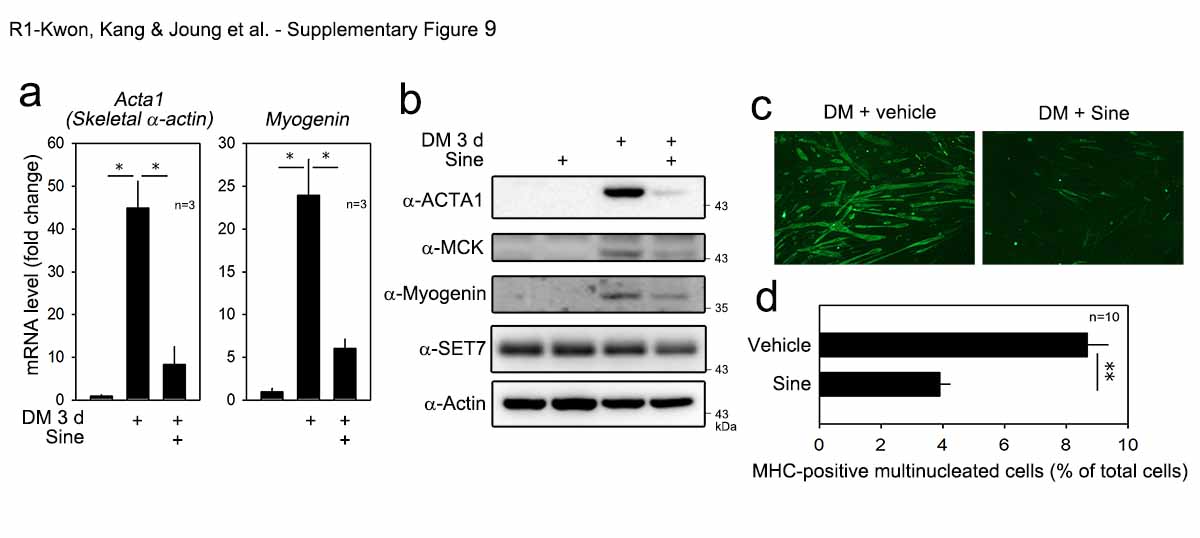
Supplementary Fig S9. Sinefungin (sine) attenuates skeletal muscle cell differentiation.** a. sine attenuates the mRNA levels of Acta1 (left) and Myogenin (right) increased by treatment with DM for 3 d. b. sine decreases the protein expression of ACTA1, MHC, and Myogenin. c. Immunocytochemical image of MHC expression. d. Multinucleated cell counts. *p < 0.05; **p < 0.01
